# Is gap balancing superior to measured resection technique in total knee arthroplasty? A meta-analysis

**DOI:** 10.1186/s42836-020-0025-1

**Published:** 2020-01-29

**Authors:** Qiang He, Caihong Sun, Jianbing Ma, Jianbing Guo

**Affiliations:** 1 Department of Orthopaedic Surgery, Hong-Hui Hospital, Xi’an Jiaotong University Health Science Center, No. 555 East Youyi Road, Xi’an, 710054 China; 2 Department of Informatics, 451 Hospital of PLA, No. 269 East Youyi Road, Xi’an, 710054 China

**Keywords:** Total knee arthroplasty, Measured resection, Gap balancing

## Abstract

**Background:**

Measured resection and gap balancing are two distinct methods for proper femoral component alignment in total knee arthroplasty. Decision-making between the two techniques is controversial. The aim of this systematic review and meta-analysis was to compare measured resection and gap balancing with regard to the radiological and clinical benefits, and to examine whether this change the conclusions from previous trails.

**Methods:**

A systematic literature search of the medical literature from January 1990 to February 2015 was performed. We selected six randomized controlled trials and five prospective cohort studies comparing gap balancing and measured resection in patients undergoing primary total knee arthroplasty. Data from included studies were pooled with use of fixed-effects and random-effects models with standard mean differences and risk ratios for continuous and dichotomous variables, respectively. Heterogeneity across studies was assessed with calculation of the I^2^ statistic.

**Results:**

A total of 857 knees from 11 trials were included. Four hundred and forty-one knees were treated with gap balancing and 416 were treated with measured resection. In contrast to previous studies, we found that gap balancing demonstrated better patient-reported outcomes with regard to Knee Society score for pain (WMD 2.75, *p* = 0.004) and Knee Society score for function (WMD 5.47, *p* < 0.0001) at two-year follow-up. Gap balancing showed more precise limb alignment in terms of post-operative value of mechanical axis (WMD 0.40°, *p* = 0.01) and risk of mechanical alignment outliers (RR 0.350, *p* < 0.0001). However, gap balancing was associated with more joint line elevation (WMD 1.27 mm, *p* < 0.0001) and longer operative time (WMD 16.18 min, *p* < 0.0001). No significant difference was observed in rotation of the femoral component (*p* = 0.07).

**Conclusions:**

The meta-analysis demonstrated that gap balancing was able to achieve more precise coronal alignment with better short-term patient-reported outcomes compared with measured resection. Measured resection was more desirable than gap balancing with regard to restoration of the joint line and operative time. Comparable femoral rotational alignment was observed.

## Background

Total knee arthroplasty (TKA) has shown to portend good long-term survivorship and excellent patient satisfaction [1–4]. However, about 6 % of primary TKAs fail within 10 years and require revision surgery, of which incorrect soft tissue balancing is a major cause [5]. There are two distinct methods for proper femoral component alignment reported in the literature: measured resection (MR) and gap balancing (GB) [6]. In the measured resection technique, bone landmarks are used to guide resections equal to the distal and posterior thickness of the femoral component. In the gap balancing technique, equal collateral ligament tension in flexion and extension is used as a guide to final bone cuts [7].

Decision-making regarding gap balancing versus measured resection during TKA is controversial. Most of the literature compares femoral component rotation and kinematics [4, 8, 9]. Patient-reported outcome is an important aspect of clinical decision-making during TKA [10]. Previous studies have compared patient-reported outcomes between the two techniques, and in those series, no difference has been observed [11–13]. However, the interpretation in those series was limited by inclusion of participants with discrepant pathologies and small sample sizes of participants [13]. Two systematic reviews of gap balancing versus measured resection have been published [6, 7]. However, the two reviews based their conclusions primarily on descriptive summaries, included observational studies and did not perform meta-analyses. Moreover, since the publication of those studies, several randomized controlled trials have been conducted and published [14–16].

For these reasons, we performed a systematic literature review and meta-analysis to derive a more precise estimation of the comparison between gap balancing and measured resection technique. We hypothesized that gap balancing would be associated with better overall patient-reported outcomes. Utilizing pooled data and meta-analysis, we compared gap balancing with measured resection in terms of radiological and clinical benefits as indicated by the currently available evidence.

## Methods

### Literature review

Eligible studies for this meta-analysis were randomized controlled trials (RCTs) or prospective cohort studies comparing gap balancing with measured resection in terms of radiological and/or clinical outcomes. Inclusion criteria included reports comparing gap balancing and measured resection technique in patients undergoing primary TKAs, RCTs or prospective cohort studies, and English-language publication. Exclusion criteria included animal or cadaver studies. The most commonly used medical databases (Embase, Medline, Cochrane Central Register of Controlled Trials, and Web of Science) from January 1990 through February 2015 were queried with use of the terms “gap balancing technique” OR “gap-balancing” OR “gap-balancing technique” OR “gap technique” AND “measured resection” OR “measured resection technique” OR “measured-resection”. These queries returned 37, 38, 35 and 48 results from the four databases respectively. Two reviewers also scanned the reference lists of the included articles for additional articles that met the inclusion criteria, and searched relevant meetings from 1990 to 2015 to identify unpublished reports. The quality of each study selected for inclusion was evaluated by two independent reviewers with the PEDro critical appraisal tool. This 11-item critical appraisal tool is designed to evaluate comparability between the groups, method of randomization, blinding and statistical analysis of RCTs. This instrument has previously demonstrated reliability and validity [17].

### Study design

Eleven studies met the inclusion criteria and were the focus of the present study. The studies were published between 2010 and 2014. Six of the studies represented Level-I evidence (RCTs), and five studies represented Level- II evidence (prospective cohort studies). Data were extracted from each eligible study using a standard form. Information retrieved from each study included authors, year of publication, study design, patient gender, mean age, sample size, prosthesis design, radiological and clinical outcomes and follow-up period. When necessary, attempts were made to contact the authors of the included studies to clarify reported data or to obtain missing data.

The primary outcome evaluated for this study was patient-reported outcomes, including Knee Society score (KSS) and Functional Knee Society score (FKSS). The secondary outcomes included: rotation of the femoral component, mechanical alignment, risk of mechanical alignment outliers, joint line elevation, operative time and complications. Rotation of the femoral component was defined as the angle between the surgical transepicondylar axis and the posterior condylar line on the axial post-operative CT images [8]. Mechanical alignment was defined as the angle between the mechanical axis of the femur and the mechanical axis of the tibia on the post-operative full-leg radiographs [18]. Mechanical alignment outlier was defined as post-operative value of the mechanical axis exceeding the ideal value of 180° by more than 3° [19]. Joint line elevation was defined as the difference between preoperative joint line and postoperative joint line [20]. Complications included: deep or superficial infection, deep vein thrombosis and stiff knee. Any discrepancies in data extraction between reviewers were resolved by mutual agreement.

### Statistical analysis

All meta-analyses were performed with the Stata software (version10.0, StataCorp, Texas, USA) according to Cochrane Collaboration and Quality of Reporting of Meta-analyses guidelines. For continuous data, such as KSS and mechanical alignment, means and standard deviation (SD) were used to calculate a weighted mean difference (WMD) and 95% confidence interval (CI). For dichotomous data, such as mechanical alignment outliers, the risk ratio (RR) and 95% CI were calculated as the summary test. Pooled summary tests were calculated with use of a fixed-effect model if heterogeneity was not significant or with use of a random effect model if heterogeneity was significant. A *P* value on I^2^ test of less than 0.10 was considered to be evidence of heterogeneity [21]. Sensitivity analyses were performed by sequentially leaving one study out to assess the consistency of results whenever possible. In addition, we assessed the impact of the quality of the study on the outcome, with a subgroup analysis performed in which randomized controlled trials were compared with prospective cohort studies. A probability of *p* < 0.05 was considered to be statistically significant.

## Results

**Fig. 1:**
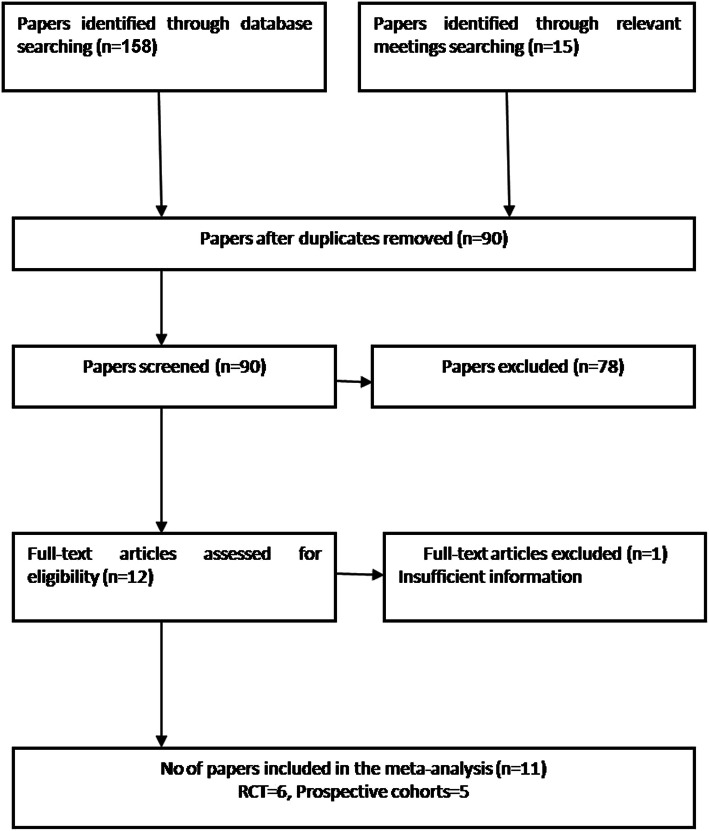
Study flow diagram of the system review

A total of 158 potentially relevant studies were obtained from searches of the various electronic bibliographies. A further 15 papers were identified from relevant meetings. Twelve studies satisfied the eligibility criteria and were included in the review [8, 9, 11–13, 15, 16, 18–20, 22, 23]. Of these studies, the trial by Jong and colleagues with 64 patients was excluded, because it was not possible to extract sufficient information from the reported data [22]. Therefore, we included 11 papers involving 857 knees (Fig. 1). Table 1 summarizes major characteristics of included studies. Four hundred and forty-one knees were treated with gap balancing and 416 were treated with measured resection. The patients included were predominantly female, and majority of antecedent pathologies were osteoarthritis. The minimum duration of follow-up reporting patient-reported outcomes was 2 years. The quality of these studies was evaluated with PEDro critical appraisal score and the exact outcome is summarized in the Table 2. This indicated that there was considerable variability in the evidence base. Six RCTs and five prospective cohort studies were identified. Of these, only three concealed the randomization procedure adequately [11, 15, 24]. Whilst surgeon blinding might have been inappropriate in these studies, seven studies did not blind their assessors to patient group [8, 9, 13, 14, 19, 20, 23]. The authors of five of the 11 studies clearly stated that their analysis was based on intention-to-treat principles [9, 11, 13, 15, 18].

**Table 1:** The major characteristics of the included trails

	Year of publication	Study design	Sample Size		Mean Age		Gender (F/M)		Pathologies (OA/RA)		Prosthesis design	Length of follow-up
			GB	MR	GB	MR	GB	MR	GB	MR		
Babazadeh [15]	2014	RCT	51	52	69.9	70.2	37/14	30/22	45/5	50/2	CR	2 years
Baier [9]	2014	RCT	19	21	72.4	70.0	14/5	12/9	19/0	21/0	CR	6 months
Nikolaides [14]	2014	Prospective cohort	29	34	70.0	71.0	NR	NR	20/0	34/0	PS	7 days
Luyckx [8]	2012	Prospective cohort	48	48	65.0	64.0	30/18	34/14	48/0	48/0	PS	NR
Singh [12]	2012	RCT	26	26	73.0	73.0	NR	NR	26/0	26/0	CR	2 years
Lee HJ [13]	2011	RCT	30	30	68.9	68.9	28/2	28/2	26/4	30/0	CR	2 years
Pang [11]	2011	RCT	70	70	68.0	70.0	60/10	58/12	70/0	70/0	CR	2 years
Sabbioni [20]	2011	Prospective cohort	31	36	67.0	69.0	25/6	28/8	31/0	36/0	CR	7 days
Lee DH [24]	2010	RCT	60	56	66.0	67.0	57/3	54/2	60/0	56/0	CR	3 months
Tigani [19]	2010	Prospective cohort	57	66	67.0	69.0	42/15	46/20	55/1	64/2	PS	7 months
Dennis [23]	2010	Prospective cohort	20	40	NR	NR	NR	NR	20/0	40/0	PS, CR	2 years

*GB* Gap balancing, *MR* Measured resection, *RCT* Randomized controlled trials, *NR* Not reported, *CR* Cruciate-retaining prostheses, *PS* Posterior cruciate-stabilizing prostheses

**Table 2:** PEDro critical appraisal score

Study	PEDro criteria											Total
	1	2	3	4	5	6	7	8	9	10	11	
Babazadeh [15]	Y	Y	Y	Y	Y	N	Y	Y	Y	Y	Y	10
Pang [11]	Y	Y	Y	Y	Y	N	Y	Y	Y	Y	Y	10
Lee DH [24]	Y	Y	Y	Y	Y	N	Y	N	Y	Y	Y	9
Baier [9]	Y	Y	N	Y	Y	N	N	Y	Y	N	Y	7
Lee HJ [13]	Y	Y	N	Y	Y	N	N	Y	Y	Y	N	7
Singh [12]	Y	Y	N	N	Y	N	Y	Y	N	Y	N	6
Nikolaides [14]	Y	N	N	Y	N	N	N	N	N	Y	Y	4
Luyckx [8]	Y	N	N	Y	N	N	N	N	N	Y	Y	4
Sabbioni [20]	N	N	N	Y	N	N	N	N	N	Y	Y	3
Tigani [19]	Y	N	N	N	N	N	N	N	N	Y	Y	3
Dennis [23]	Y	N	N	N	N	N	N	N	N	Y	N	2

*Y* Yes, *N* No

1. Eligibility criteria

2. Random allocation

3. Concealed allocation

4. Baseline comparability

5. Blind subject

6. Blind clinician

7. Blind assessor

8. Adequate follow-up

9. Intention-to-treat analysis

10. Between-group analysis

11. Point estimates and variability

### Patient-reported outcomes

Four comparative studies reported outcomes of KSS and FKSS scores [9, 11–13]. One RCT reported outcomes of six-month KSS, and one RCT reported outcomes of six-month FKSS. A total of three RCTs reported outcomes of two-year KSS and FKSS, with 126 patients undergoing gap balancing and 126 patients undergoing measured resection. Therefore, the three RCTs were used to calculate the pooled results. Of the three RCTs, one RCT included participants with discrepant pathologies, and two other RCTs included patients uniformly with osteoarthritis. We excluded the trial with discrepant pathologies to conduct sensitivity analyses.

**Fig. 2:**
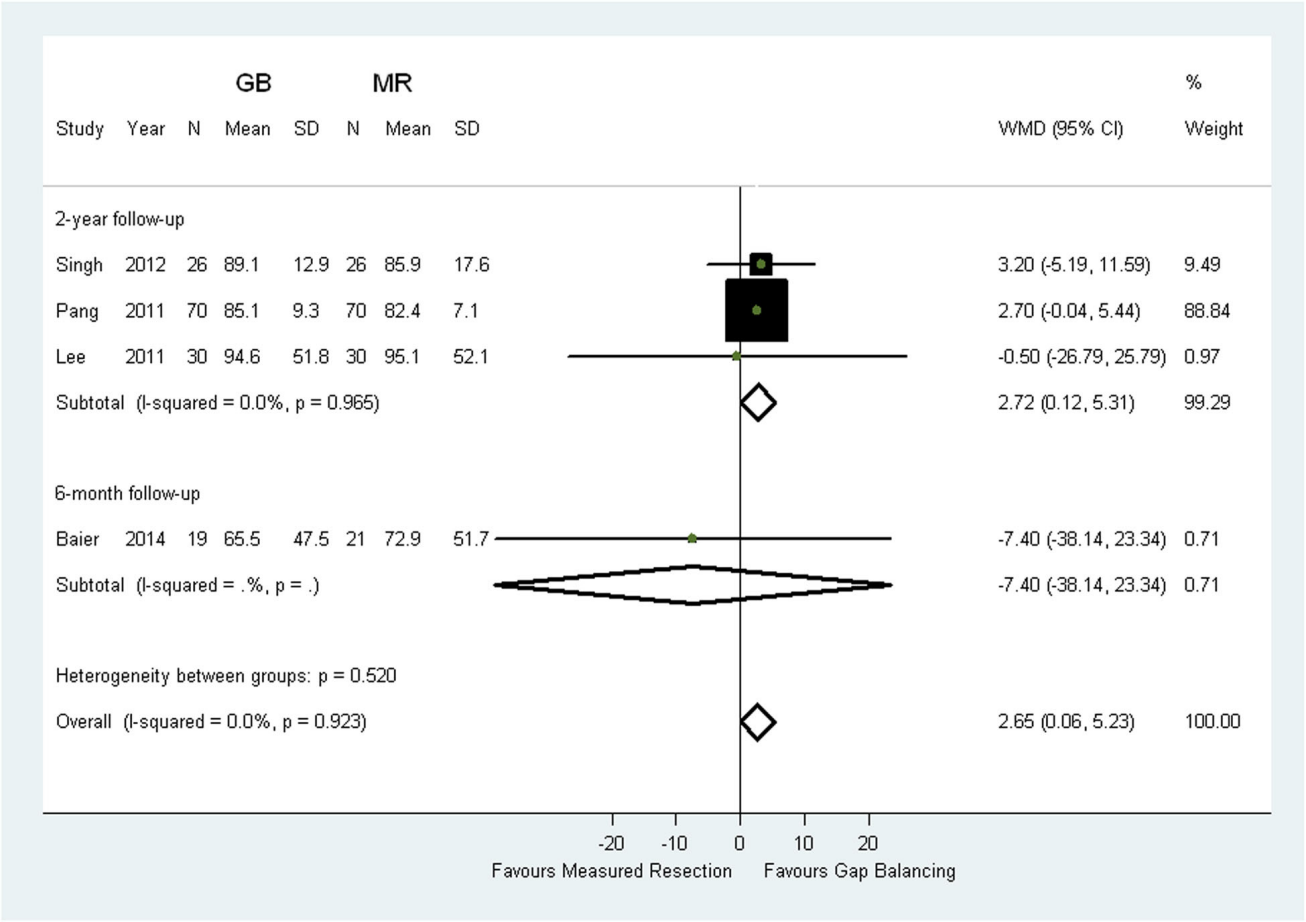
Comparison of KSS scores for pain between measured resection (MR) group and gap balancing (GB) group. SD = standard deviation, CI = confidence interval

**Fig. 3:**
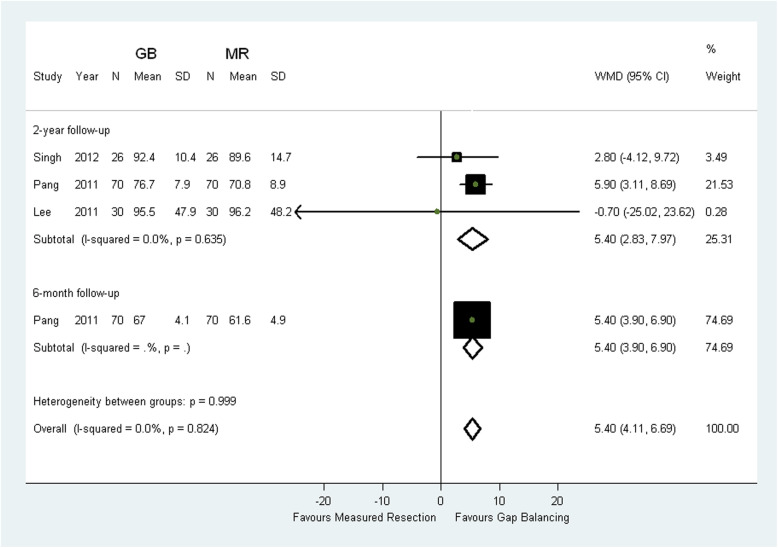
Comparison of KSS scores for function between measured resection (MR) group and gap balancing (GB) group. SD = standard deviation, CI = confidence interval

The meta-analysis results of two-year KSS scores for pain showed that gap balancing resulted in better patients-reported outcomes compared with measured resection (WMD 2.72; 95% CI 0.12 to 5.31; *p* = 0.004; Fig. 2). There were also higher two-year KSS scores for function in gap balancing compared with measured resection (WMD 5.40; 95% CI 2.83 to 7.97; *p* < 0.0001; Fig. 3). Sensitivity analyses excluding the trail with discrepant pathologies did not change the results (Table 3) [13]. However, there was no statistical difference between the two groups in terms of six-month KSS scores for pain (MD -7.4; 95% CI − 38.14 to 23.34; *p* = 0.06) [9] and KSS scores for function (MD 5.4; 95% CI 3.9 to 6.9; *p* < 0.0001) [11].

**Table 3:** Sensitivity analyses of knee society score and functional knee society score

Study	GB			MR			WMD (95% CI)	*P*
	N	Mean	SD	N	Mean	SD		
A Knee Society score								
All eligible studies							2.72(0.12,5.31)	0.04
Singh [12]	26	89.1	12.9	26	85.9	17.6		
Pang [11]	70	85.1	9.3	70	82.4	7.1		
Lee HJ [13]	30	94.6	51.8	30	95.1	52.1		
Excluding the study with different antecedent pathologies							2.75(0.14, 5.35)	0.04
Singh [12]	26	89.1	12.9	26	85.9	17.6		
Pang [11]	70	85.1	9.3	70	82.4	7.1		
B Functional Knee Society score								
All eligible studies							5.40(2.83,7.97)	< 0.0001
Singh [12]	26	92.4	10.4	26	89.6	14.7		
Pang [11]	70	76.7	7.9	26	70.8	8.9		
Lee HJ [13]	30	95.5	47.9	30	96.2	48.2		
Excluding the study with different antecedent pathologies							5.47(2.88,8.05)	< 0.0001
Singh [12]	26	92.4	10.4	26	89.6	14.7		
Pang [11]	70	76.7	7.9	26	70.8	8.9		

*GB* Gap balancing, *MR* Measured resection

### Rotation of the femoral component

A total of three studies [8, 14, 15], including one RCT [15] and two prospective cohort studies [8, 14], reported outcomes for rotation of the femoral component, with a total of 128 patients undergoing gap balancing and 134 undergoing measured resection. Subgroup analysis of femoral component rotation by study design was also conducted. For RCTs, gap balancing resulted in more external rotation than measured resection (WMD 1.00°; 95% CI 0.031 to 1.969°; *p* = 0.04). For prospective cohort studies, no significant difference was found between gap balancing and measured resection (WMD 0.27°; 95% CI − 0.470 to 1.010°; *p* = 0.48; I^2^ = 57.0%). Results of overall effect showed no statistically significant difference between the two groups (WMD 0.54°; 95% CI − 0.05 to 1.13°; *p* = 0.07; I^2^ = 46.0%; Table 4).

**Table 4:** Subgroup analysis of radiological outcomes

Radiological outcomes by study design	Groups (n)		Overall effect		*P* value	I^2^
	GB	MR	Effect estimate	95% CI		
Rotation of the femoral component	128	134	0.54°	−0.05-1.13°	0.073	46.0%
RCTs [15]	48	48	1.00°	0.031–1.969°	*0.043*	NE
Prospective cohort [8, 14]	80	86	0.27°	−0.470 -1.010°	0.475	57.0%
Post-operative value of mechanical axis	235	240	0.40°	0.10–0.71°	*0.01*	41.6%
RCTs [11, 18]	130	126	0.68°	0.26–1.11°	*0.002*	0.0%
Prospective cohort [8, 19]	105	114	0.11°	−0.32- 0.55°	0.607	18.2%
Risk of mechanical alignment outlier	174	155	0.350	0.19–0.63°	*< 0.0001*	0.0%
RCTs [11, 18]	130	126	0.375	0.202–0.696	*0.002*	0.0%
Prospective cohort [19]	57	66	0.193	0.024–1.556	0.122	NE
Joint line elevation	138	148	1.27 mm	1.64–1.96 mm	*< 0.0001*	0.0%
RCTs [13, 15]	81	82	1.319 mm	0.905–1.732 mm	*< 0.0001*	0.0%
Prospective cohort [19]	57	66	1.100 mm	0.285–1.915 mm	*0.008*	NE

*GB* Gap balancing, *MR* Measured resection, *NE* Not estimatable, Entries in italic were considered as statistically significant

### Post-operative value of mechanical axis

A total of four studies [8, 11, 18, 19], including two RCTs [11, 18] and two prospective cohort studies [8, 19], reported outcomes for post-operative value of mechanical axis, with a total of 235 patients undergoing gap balancing and 240 undergoing measured resection. Subgroup analysis by study design revealed that gap balancing resulted in more accurate alignment of mechanical axis in RCTs (WMD 0.68°; 95% CI 0.26–1.11°; *p* = 0.002; I^2^ = 0%), but not in prospective cohort studies (WMD 0.11°; 95% CI -0.32-0.55°; *p* = 0.61; I^2^ = 18.2%). Results of overall effect indicated that gap balancing had better alignment of mechanical axis (WMD 0.40°; 95% CI 0.10–0.71°; *p* = 0.01; I^2^ = 41.6%; Table 4).

### Risk of mechanical alignment outliers

A total of three studies [11, 18, 19], including two RCTs [11, 18] and one prospective cohort study [19], reported outcomes for risk of mechanical alignment outliers, with a total of 174 patients undergoing gap balancing and 155 undergoing measured resection. Subgroup analysis revealed that significant lower risk of outliers in gap balancing was found in RCTs (RR 0.375; 95% CI 0.202–0.696; *p* = 0.002; I^2^ = 0.0%) and in prospective cohort study (RR 0.193; 95% CI 0.024–1.556; *p* = 0.122) separately. The overall likelihood of mechanical alignment outliers was significantly lower with the gap balancing than with the measured resection (RR 0.350; 95% CI 0.19–0.63; *p* < 0.0001; I^2^ = 0.0%; Table 4).

### Joint line elevation

Two RCTs [13, 15] and one prospective cohort study [19] comprising a total of 138 patients undergoing gap balancing and 148 undergoing measured resection reported outcomes for joint line elevation. Subgroup analysis indicated that gap balancing resulted in more joint line elevation in RCTs (WMD 1.319 mm; 95% CI 0.905–1.732 mm; *p* < 0.0001; I^2^ = 0.0%) and in prospective cohort study (WMD 1.100 mm; 95% CI 0.285–1.915 mm; *p* = 0.008) respectively. Results of overall effect indicated that gap balancing was associated with significantly more joint line elevation (WMD 1.27 mm; 95% CI 1.64–1.96 mm; *p* < 0.0001; I^2^ = 0.0%; Table 4).

### Operative time

**Fig. 4:**
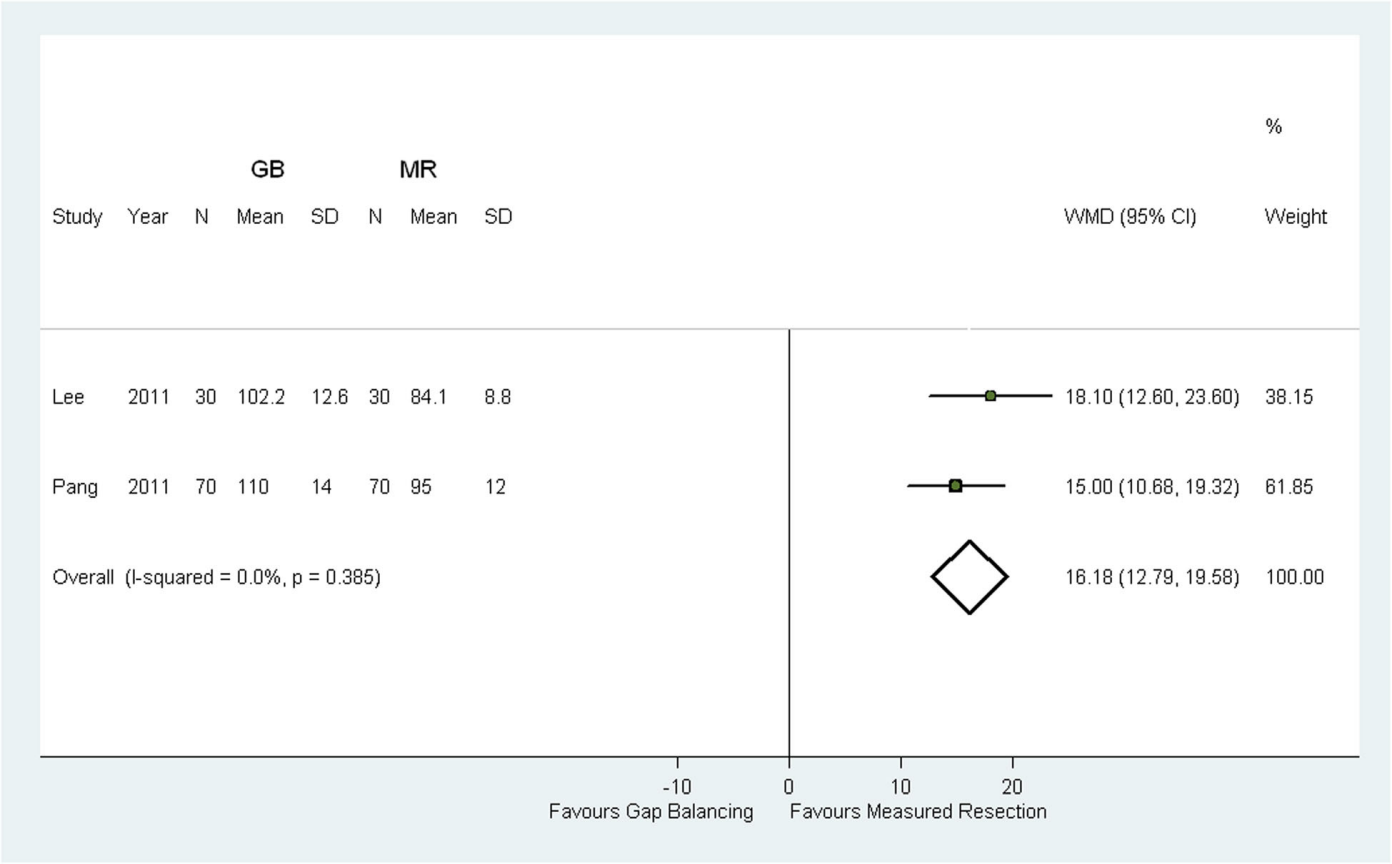
Comparison of operative time between measured resection (MR) group and gap balancing (GB) group. SD = standard deviation, CI = confidence interval

Two RCTs [11, 13] comprising a total of 100 patients undergoing gap balancing and 100 undergoing measured resection, and no prospective cohort study, reported outcomes for operative time. The operative time was significantly longer in the gap-balancing group (WMD 16.18 min; 95% CI 12.79–19.58 min; *p* < 0.0001; Fig. 4).

### Complications

Only one RCT [11] comprising 70 patients undergoing gap balancing and 70 undergoing measured resection, reported the rate of complications. Complications included pin-track or superficial wound infection (1.4%), deep vein thrombosis (1.4%) and stiff knee (1.4%). The results indicated that there was no difference between the two groups with respect to the incidence of complications.

## Discussion

The most important finding of this meta-analysis is that gap balancing was found to be superior to measured resection in regard to overall patient-reported outcomes at two-year follow-up. The KSS with gap balancing was 2.75 points higher than that obtained with measured resection (*p* = 0.004). The FKSS with gap balancing was 5.47 points higher than that obtained with measured resection (*p* < 0.0001). The results supported the hypothesis that gap balancing was associated with better overall patient-reported outcomes. Nonetheless, difference of 2.75 points in KSS and 5.47 points in FKSS may not be considered clinically meaningful [25–27]. In contrast, previous comparative studies indicated no statistical differences in KSS and FKSS between gap balancing and measured resection [9, 11–13, 28, 29]. As Table 1 demonstrates, those outcomes were measured in a small number of subjects. Accordingly, the conclusion in previous studies might be attributed to type II statistical error [30]. Although the moderate sample-size was appropriate to address project aims, larger cohorts might better detect clinically important differences [31]. In our meta-analysis, the large patient cohort (*n* = 192) provides sufficient statistical power to show differences in patient-reported outcomes between the two surgical strategies. Importantly, we observed no heterogeneity across trials for KSS or FKSS at two-year follow-up (I^2^ = 0%). Different pathologies were reported to affect patient who reported pain with TKA [32]. It was reported that significant improvements were observed with greater impact in OA than RA after TKA [32, 33]. In this meta-analysis, we excluded the trial with different antecedent pathologies [13]. Sensitivity analyses limited to cohorts with uniform pathologies, however, yielded similar results (Table 3). Other meta-analysis also concluded that GB techniques were associated with Knee Society Scores and Knee Society Function scores. In a meta-analysis, eight randomized controlled trials were included. The meta-analysis showed that GB techniques resulted in statistically significant improvements in the restoration of mechanical and rotational alignment and mean Knee Society Scores and Knee Society Function scores 2 years post-operatively, but resulted in greater elevation of the position of the joint line [34]. Another meta-analysis conducted by Li S included 20 studies involving 2259 cases. The study showed that The GB technique was associated with statistically significant increases in the primary outcomes of KSS-function in 1 year. However, a mean difference of 2.12 points was below the minimal clinically important difference of 6 points. Secondary outcome assessments showed significantly decreased surgical time (mean difference, 16.18; *P* < .00001) for MR. Although statistically significant difference in favor of GB was identified in total outliers (risk ratio, 1.72, *P* = .0004), the 2 techniques were comparable in range of motion, Western Ontario and McMaster University Osteoarthritis Index, femoral component rotation, complications, and revision rate [35].

Comparable femoral rotational alignment was recorded in all three studies pooled for rotation of the femoral component [8, 14, 15]. However, results for rotation of the femoral component showed a higher level of heterogeneity (I^2^ = 57%) in non-randomized studies. There was wide variability in pre-operative mean rotational alignment (0.8° internal rotation in GB and 1.2° internal rotation in MR in the study by Luyckx * et al*.; 5.9° external rotation in GB and 6.4° external rotation in MR in the study by Nikolaides *et al*.) in the two prospective cohort studies [8, 14]. We considered the physiological variability in pre-operative mean rotational alignment to be the main factors in this large variation among studies generating substantial heterogeneity. A recent study showed that, with the measured resection technique with preoperative CT, the femoral component was externally rotated approximately 1 degree more than in the gap balancing or measured resection technique without preoperative CT [36]. GB techniques seem to be associated with more external rotation in femoral rotational alignment.

Gap balancing was able to achieve more precise coronal alignment. Post-operative values of mechanical axis were pooled from four studies [8, 11, 18, 19], and we identified the non-randomized studies (I^2^ = 18%) to be the most important source of heterogeneity. The most plausible reason for this heterogeneity is that the surgical methods were different between the two non-randomized studies (computer-assisted surgery *versus* conventional technique) [8, 19]. Computer-assisted surgery has been proved to improve the accuracy of implant positioning and extremity alignment [37]. Of the four studies, only the trail by Luyckx *et al*. performed TKA with conventional technique [8]. Risk of mechanical alignment outlier was pooled from three studies [11, 18, 19], and showed no heterogeneity across trails (I^2^ = 0%). Gap balancing was able to demonstrate more accurate limb alignment with fewer outliers than measured resection. Participants who underwent measured resection experienced a 2.9-fold increased risk of mechanical alignment outliers compared with gap balancing.

There are some disadvantages with gap balancing technique, including joint line elevation and protracted time of surgery. For joint line elevation, the results were more robust: estimates for both RCTs and prospective cohort studies pointed in the same direction and no heterogeneity was found across trails (I^2^ = 0%) [13, 15, 19]. Gap balancing resulted in 1.27 mm more joint line elevation on average compared with measured resection. The raised joint line with gap balancing can be attributed to this technique’s prioritization of gap symmetry [6, 38, 39]. Although not included in the meta-analysis, measured resection was found to be two-fold risky to suffer femoral condylar lift-off compared with gap balancing [23]. A significant disadvantage with gap balancing was found to be operative time [13, 25]. Gap balancing resulted in increased operative time by 16 min on average compared with measured resection. With gap balancing technique, surgeons operate with spacer blocks or ligament tensioners to obtain rectangular gap, which would need some extra time [16, 40, 41]. However, since we were unable to distinguish the results between experienced and inexperienced surgeons, it remained unclear whether this result can be ascribed to the increased operative time with gap balancing.

Recently, Daines *et al*. performed a systematic review of gap balancing *versus* measured resection technique in TKA [6]. The authors concluded that while measured resection techniques can be accurate in a majority of cases, exclusive use of this technique often results in flexion gap asymmetry and an increased incidence of femoral condylar lift-off. The gap balancing technique is less dependent on bony anatomy and can be used to provide more reproducible flexion gap stability [6]. The review by Springer *et al*. indicated that neither the gap balancing technique nor measured resection technique is definitively better than the other [7]. However, since the publication of those studies, several randomized controlled trials have been conducted and published. In our study, we focused on clinical outcomes and used patient-reported outcomes as a primary end point.

To our acknowledgement, this is the first meta-analysis in the literature comparing gap balancing and measured resection technique. Furthermore, all authors were contacted to clarify areas of uncertainty and to provide unreported data, which consisted of unreported standard deviations. We successfully obtained required raw data of patient-reported outcomes, making it possible to pool comparable outcome measurements from more studies and therefore to augment the quantity of evidence.

This meta-analysis has several limitations. The 11 included trails preformed TKA for a variety of diagnoses with variable durations of follow-up and variable outcome measures. Acceptable trials comparing gap balancing with measured resection in regard to patient-reported outcomes were limited to four studies. However, the large patient cohort was sufficient to find differences in patient-reported outcomes between the two groups. Second, moderate heterogeneity was observed in several secondary outcome measurements. Nonetheless, we were able to identify important heterogeneity sources such as different surgical methods and large physiological variations. We believed the pooling of comparable outcomes and the identification of sources of heterogeneity are the best option for meta-analysis. However, conclusions from these substantially heterogeneous outcomes should be interpreted with caution.

## Conclusions

In summary, this meta-analysis indicated that gap balancing was able to achieve more precise coronal alignment with better short-term patient-reported outcomes compared with measured resection. The gap balancing and measured resection technique achieved comparable restoration of femoral rotational alignment. It should be taken into account that there are some drawbacks with gap balancing technique, including joint line elevation and prolonged time of surgery. Since KSS and WOMAC were the most common outcome instruments used in clinical trials of knee replacement, future randomized trials with the three outcome instruments are needed to define the patient-reported outcomes between the two techniques. Furthermore, the difference in long-term patient-reported outcomes between the two techniques should be determined. Finally, the two surgical techniques both have important advantages and disadvantages. Another area for future research is the study of criteria that will allow physicians to select patients who are appropriate for gap balancing or measured resection technique.

## Data Availability

Not applicable

## Abbreviations

GB: Gap balancing
MR: Measured resection
RCTs: Randomized controlled trials
TKA: Total knee arthroplasty

## References

[1] Thienpont E, Opsomer G, Koninckx A, Houssiau F (2014). Joint awareness in different types of knee arthroplasty evaluated with the forgotten joint score. J Arthroplast.

[2] Shan L, Shan B, Suzuki A, Nouh F, Saxena A (2015). Intermediate and long-term quality of life after total knee replacement: a systematic review and meta-analysis. J Bone Joint Surg Am.

[3] Jauregui JJ, Cherian JJ, Pierce TP, Beaver WB, Issa K, Mont MA. Long-term survivorship and clinical outcomes following total knee arthroplasty. J Arthroplasty. 2015; Epub 2015/06/24. 10.1016/j.arth.2015.05.052 26100473

[4] Myers TG, Cui Q, Kuskowski M, Mihalko WM, Saleh KJ (2006). Outcomes of total and unicompartmental knee arthroplasty for secondary and spontaneous osteonecrosis of the knee. J Bone Joint Surg Am.

[5] McNabb DC, Kim RH, Springer BD (2015). Instability after total knee arthroplasty. J Knee Surg.

[6] Daines BK, Dennis DA (2014). Gap balancing vs. measured resection technique in total knee arthroplasty. Clin Orthop Surg.

[7] Springer BD, Parratte S, Abdel MP (2014). Measured resection versus gap balancing for total knee arthroplasty. Clin Orthop Relat Res.

[8] Luyckx T, Peeters T, Vandenneucker H, Victor J, Bellemans J (2012). Is adapted measured resection superior to gap-balancing in determining femoral component rotation in total knee replacement?. J Bone Joint Surg Br.

[9] Baier C, Fitz W, Craiovan B, Keshmiri A, Winkler S, Springorum R (2014). Improved kinematics of total knee replacement following partially navigated modified gap-balancing technique. Int Orthop.

[10] Meloni MC, Hoedemaeker RW, Violante B, Mazzola C (2014). Soft tissue balancing in total knee arthroplasty. Joints.

[11] Pang HN, Yeo SJ, Chong HC, Chin PL, Ong J, Lo NN (2011). Computer-assisted gap balancing technique improves outcome in total knee arthroplasty, compared with conventional measured resection technique. Knee Surg Sports Traumatol Arthrosc.

[12] Singh VK, Varkey R, Trehan R, Kamat Y, Raghavan R, Adhikari A (2012). Functional outcome after computer-assisted total knee arthroplasty using measured resection versus gap balancing techniques: a randomised controlled study. J Orthop Surg (Hong Kong).

[13] Lee HJ, Lee JS, Jung HJ, Song KS, Yang JJ, Park CW (2011). Comparison of joint line position changes after primary bilateral total knee arthroplasty performed using the navigation-assisted measured gap resection or gap balancing techniques. Knee Surg Sports Traumatol Arthrosc.

[14] Nikolaides AP, Kenanidis EI, Papavasiliou KA, Sayegh FE, Tsitouridis I, Kapetanos GA (2014). Measured resection versus gap balancing technique for femoral rotational alignment: a prospective study. J Orthop Surg (Hong Kong).

[15] Babazadeh S, Dowsey MM, Stoney JD, Choong PF (2014). Gap balancing sacrifices joint-line maintenance to improve gap symmetry: a randomized controlled trial comparing gap balancing and measured resection. J Arthroplast.

[16] Matsumoto T, Muratsu H, Kawakami Y, Takayama K, Ishida K, Matsushita T (2014). Soft-tissue balancing in total knee arthroplasty: cruciate-retaining versus posterior-stabilised, and measured-resection versus gap technique. Int Orthop.

[17] Maher CG, Sherrington C, Herbert RD, Moseley AM, Elkins M (2003). Reliability of the PEDro scale for rating quality of randomized controlled trials. Phys Ther.

[18] Lee DH, Park JH, Song DI, Padhy D, Jeong WK, Han SB (2010). Accuracy of soft tissue balancing in TKA: comparison between navigation-assisted gap balancing and conventional measured resection. Knee Surg Sports Traumatol Arthrosc.

[19] Tigani D, Sabbioni G, Ben Ayad R, Filanti M, Rani N, Del Piccolo N (2010). Comparison between two computer-assisted total knee arthroplasty: gap-balancing versus measured resection technique. Knee Surg Sports Traumatol Arthrosc.

[20] Sabbioni G, Rani N, Del Piccolo N, Ben Ayad R, Carubbi C, Tigani D (2011). Gap balancing versus measured resection technique using a mobile-bearing prosthesis in computer-assisted surgery. Musculoskelet Surg.

[21] Mazziotti G, Biagioli E, Maffezzoni F, Spinello M, Serra V, Maroldi R (2015). Bone turnover, bone mineral density, and fracture risk in acromegaly: a meta-analysis. J Clin Endocrinol Metab.

[22] Seon JK. Comparative study of radiological and functional outcome after TKA using the measured resection and balanced gap technique. In: 9th biennial congress of the international society of arthroscopy, knee surgery, and orthopaedic sports medicine. 2013. p. e172–e3.

[23] Dennis DA, Komistek RD, Kim RH, Sharma A (2010). Gap balancing versus measured resection technique for total knee arthroplasty. Clin Orthop Relat Res.

[24] Lee DH, Lee DK, Shin YS, Han SB (2013). Influence of gap balance on the sagittal movement of a specific mobile bearing floating platform design in total knee arthroplasty. J Arthroplast.

[25] Singh J, Sloan JA, Johanson NA (2010). Challenges with health-related quality of life assessment in arthroplasty patients: problems and solutions. J Am Acad Orthop Surg.

[26] Scuderi GR, Sikorskii A, Bourne RB, Lonner JH, Benjamin JB, Noble PC. The knee society short form reduces respondent burden in the assessment of patient-reported outcomes. Clin Orthop Relat Res. 2015; Epub 2015/06/07. 10.1007/s11999-015-4370-2 PMC4686526 26047645

[27] Liddle AD, Pandit H, Judge A, Murray DW (2015). Patient-reported outcomes after total and unicompartmental knee arthroplasty: a study of 14,076 matched patients from the National Joint Registry for England and Wales. Bone Joint J.

[28] Hernandez-Hermoso JA, Nescolarde-Selva L, Rodriguez-Montserrat D, Martinez-Pastor JC, Garcia-Oltra E, Lopez-Marne S. Different femoral rotation with navigated flexion-gap balanced or measured resection in total knee arthroplasty does not lead to different clinical outcomes. Knee Surg Sports Traumatol Arthrosc. 2019; Epub 2019/07/05. 10.1007/s00167-019-05591-3 31270588

[29] Churchill JL, Khlopas A, Sultan AA, Harwin SF, Mont MA (2018). Gap-balancing versus measured resection technique in total knee arthroplasty: a comparison study. J Knee Surg.

[30] Bland M. An introduction to medical statistics. 3rd ed. Oxford: Oxford University Press; 2006.

[31] Friedman LFFC, DeMets DL. Fundamentals of clinical trials. 3rd ed. New York: Springer; 1998.

[32] Dusad A, Pedro S, Mikuls TR, Hartman CW, Garvin KL, O'Dell JR, et al. Impact of total knee arthroplasty using patient reported pain and health-related quality of life indices: rheumatoid arthritis versus osteoarthritis. Arthritis Rheumatol. 2015; Epub 2015/07/28. 10.1002/art.39221 26213106

[33] Dusad A, Pedro S, Mikuls TR, Hartman CW, Garvin KL, O'Dell JR (2015). Impact of Total knee arthroplasty as assessed using patient-reported pain and health-related quality of life indices: rheumatoid arthritis versus osteoarthritis. Arthritis Rheumatol.

[34] Huang T, Long Y, George D, Wang W (2017). Meta-analysis of gap balancing versus measured resection techniques in total knee arthroplasty. Bone Joint J.

[35] Li S, Luo X, Wang P, Sun H, Wang K, Sun X (2018). Clinical outcomes of gap balancing vs measured resection in total knee arthroplasty: a systematic review and meta-analysis involving 2259 subjects. J Arthroplast.

[36] Kim CW, Lee CR, Gwak HC, Kim JH, Kwon YU, Kim DY. The effects of surgical technique in total knee arthroplasty for Varus osteoarthritic knee on the rotational alignment of femoral component: gap balancing technique versus measured resection technique. J Knee Surg. 2019; Epub 2019/01/09. 10.1055/s-0038-1676766 30620985

[37] Rebal BA, Babatunde OM, Lee JH, Geller JA, Patrick DA, Macaulay W (2014). Imageless computer navigation in total knee arthroplasty provides superior short term functional outcomes: a meta-analysis. J Arthroplast.

[38] Niki Y, Harato K, Nagai K, Suda Y, Nakamura M, Matsumoto M. Effects of reduction osteotomy on gap balancing during total knee arthroplasty for severe Varus deformity. J Arthroplast. 2015; Epub 2015/08/05. 10.1016/j.arth.2015.06.061 26239234

[39] Debieux P, de Oliveira JR, Franciozi CE, Kubota MS, Granata G, Luzo MV (2014). Extension and flexion gap balancing and its correlation with alignment in navigated total knee arthroplasty. Orthopedics.

[40] Gu Y, Roth JD, Howell SM, Hull ML (2014). How frequently do four methods for mechanically aligning a total knee arthroplasty cause collateral ligament imbalance and change alignment from normal in white patients? AAOS exhibit selection. J Bone Joint Surg Am.

[41] Fitz W, Sodha S, Reichmann W, Minas T (2012). Does a modified gap-balancing technique result in medial-pivot knee kinematics in cruciate-retaining total knee arthroplasty? A pilot study. Clin Orthop Relat Res.

